# Early treatment of rituximab combined with eltrombopag for secondary thrombocytopenic purpura in rheumatoid arthritis: A case report

**DOI:** 10.1097/MD.0000000000028417

**Published:** 2022-01-14

**Authors:** Naidan Zhang, Chaixia Ji, Xiao Bao, Chengliang Yuan

**Affiliations:** a Department of Clinical Laboratory, Peoples Hospital of Deyang City, Deyang, China; b Department of Rheumatology, Peoples Hospital of Deyang City, Deyang, China.

**Keywords:** case report, immature platelet fraction, rheumatoid arthritis, secondary thrombocytopenic purpura

## Abstract

**Rationale:**

Secondary immune thrombocytopenic purpura (ITP) is also known as acquired thrombocytopenic purpura, autoimmune disease is usually one of the important causes. There are few reports about treatment of refractory thrombocytopenic purpura in rheumatoid arthritis (RA). We report a case of refractory ITP in which changes in platelet-related markers with therapeutic agents are worthy of the attention of clinicians.

**Patient concerns:**

A 69-year-old woman admitted for ecchymosis on the neck and arms for 15 days presented to our hospital. She was diagnosed with RA 5 years ago.

**Diagnosis:**

The diagnosis met the American College of Rheumatology/European League Against Rheumatism 2010 classification criteria. The disease activity score 28 (DAS-28) was 4.6, indicating that the disease activity was moderate.

**Interventions:**

Treatment with first-line therapies and second-line treatment--eltrombopag (EPAG) were ineffective. Therefore, we performed rituximab combined with a low dose of EPAG.

**Outcomes:**

The patient received 2 cycles of rituximab combined with EPAG, and reported no new petechiae on her buccal mucosa and limbs during follow-up.

**Lessons:**

This case suggests that early treatment of rituximab combined with EPAG is beneficial to patients with refractory ITP in RA. In terms of disease dynamic monitoring, immature platelet fraction (IPF) may be an auxiliary indicator for predicting efficacy, but its significance needs further study.

## 1 Introduction

Rheumatoid arthritis (RA) is a chronic autoimmune disease with insidious onset and multiarticular synovitis as its basic pathological features. Secondary thrombocytopenic purpura is also known as acquired thrombocytopenic purpura, autoimmune disease is usually one of the important causes.^[1,2]^ Therefore, treating the primary disease has always been the key to improving platelet count. As secondary thrombocytopenic purpura in RA is rare, there are few reports on the treatment of the disease and the changes of platelet count or immature platelet fraction (IPF) after drug treatment.^[3]^ We shared the diagnosis and treatment of a patient with refractory thrombocytopenic purpura in .RA

In this report, we noted that the IPF changed significantly before and after the treatment of rituximab/eltrombopag (EPAG), which may be an indicator to dynamically evaluate the effectiveness of drug therapy for secondary thrombocytopenic purpura in RA.

## 2 Case presentation

The case patient was a 69-year-old woman admitted for ecchymosis on the neck and arms for 15 days. She mainly presented with a 2 × 3 cm^2^ blood blister on the right buccal mucosa and ecchymosis on the neck and upper limbs. She was diagnosed with rheumatoid arthritis 5 years ago. Specialist examination suggested that 10 proximal interphalangeal joints were impaired and scored 5 points. The length of arthritis continued for about 6 weeks and scored 1 point. Laboratory tests indicated that C-reactive protein, erythrocyte sedimentation rate, rheumatoid factor and anti-cyclic peptide containing citrulline antibody were significantly increased, with scores of 1 and 3 respectively. The diagnosis met the American College of Rheumatology/European League Against Rheumatism 2010 classification criteria. The disease activity score 28 (DAS-28) was 4.6, indicating that the disease activity was moderate.

**Figure 1:**
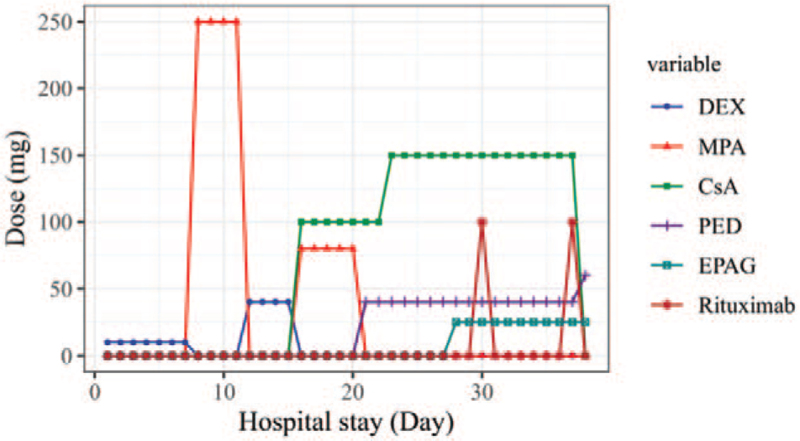
Changes in treatment and admission date. Use of anti-rheumatism drugs were represented by line graphs of different colors. Dark blue indicated DEX, red indicated MPA, light green indicated CsA, Purple indicated PED, dark green indicated EPAG, dark red indicated Rituximab. CsA = cyclosporine and platelet infusion, DEX = dexamethasone, EPAG = eltrombopag, IVIG = intravenous immunoglobulin, MPA = methylprednisolone, PED = prednisone.

**Figure 2:**
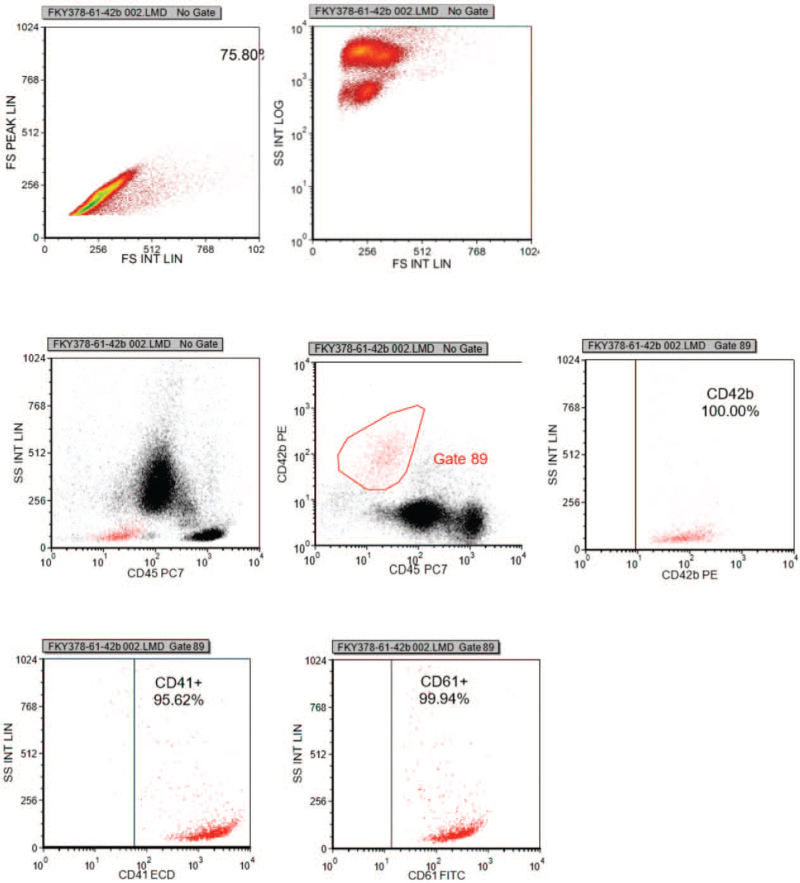
Changes in percentages of CD41, CD61, and CD42b before rituximab. Percentages of CD41, CD61, and CD42b before rituximab treatment were nomal.

After admission, she was given dexamethasone, methylprednisolone, intravenous immunoglobulin (IVIG), cyclosporine and platelet infusion (Fig. 1). The patient's blood blister had crusted, but the ecchymosis on the neck and arms did not subside. At the same time, ecchymosis increased on her left temporal and hip. Bone marrow smear indicates scattered thrombocytopenia. The platelet count was 9 × 10^9^/L, and the IPF was 33.9%. Flow cytometry showed that percentages of CD41, CD61 and CD42b were normal (Fig. 2).

**Figure 3:**
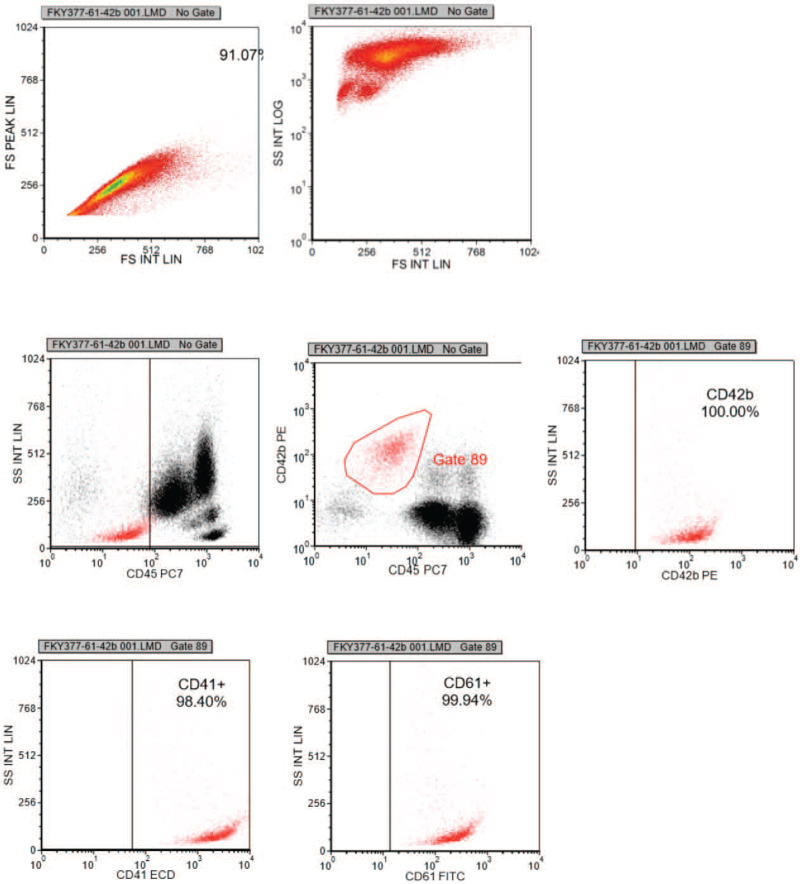
Changes in percentages of CD41, CD61, and CD42b after rituximab. Percentages of CD41, CD61, and CD42b after rituximab treatment were nomal.

**Figure 4:**
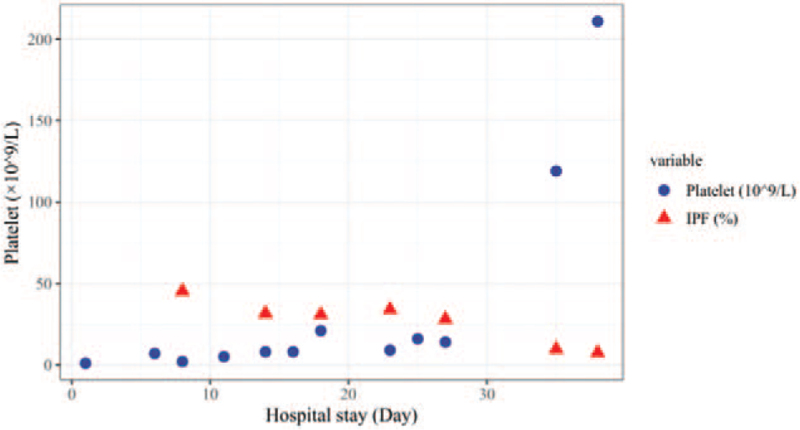
Changes of platelet and IPF with treatment. Dark blue indicated platelet, red indicated IPF. IPF = immature platelet fraction.

On September 16, 2021, the patient started to take EPAG 25 mg daily for 3 days. On September 18, 2021, the scope of ecchymosis was significantly reduced. The platelet count was 14 × 10^9^/L, and the IPF was 28.0%. It was suggested that the use of low dose EPAG alone was not effective in increasing platelet count. On the same day, she received the first course of rituximab 100 mg, and she continued with oral EPAG 25 mg daily which was well tolerated. On September 25, 2021, the platelet count was 119 × 10^9^/L, and the IPF was 9.6%. Her bruises had basically faded. Continue to give the second cycle of rituximab 100 mg combined with EPAG 25 mg, ciclosporin 150 mg and prednisone 40 mg daily for 3 days. On September 28, 2021, the platelet count was 211 × 10^9^/L, and the IPF was 7.4%. Flow cytometry showed that there was no significant difference in CD41, CD61 and CD42b after rituximab treatment compared with before (Fig. 3). The DAS-28 was 3.2. Her symptoms improved and she was discharged. She continued to receive prednisone 60 mg daily for 14 days. After 2 weeks, the patients reported no new petechiae on her buccal mucosa and limbs during telephone follow-up. The changes of platelet count and IPF with treatment regimen were shown (Fig. 4). These results suggested that the platelet formation ability in bone marrow was significantly improved with the treatment of rituximab combined with EPAG. Although there was no significant change in platelet flow cytometry, there was a significant change in IPF before and after rituximab/EPAG treatment, which was consistent with improvement in clinical symptoms.

Ethics approval for the study was obtained from Peoples Hospital of Deyang City, Deyang, China. The patient included in the study provided written informed consent. Details that might disclose the identities of the patient were removed.

## 3 Discussion and conclusions

Immune thrombocytopenic purpura (ITP) is also known as idiopathic blood small-plate purpura. Recently, it has been officially unified by the international ITP working group.^[4]^ At present, the diagnosis of ITP is still an exclusion diagnosis, and the international ITP working group recommends that the diagnosis of ITP be divided into primary and secondary.^[5]^ Secondary refers to thrombocytopenia caused by other diseases or drugs. Recent reports confirm that autoimmune diseases, patients with low cellular immune function, viral infections, and the use of drugs such as quinine and heparin are important causations of ITP.^[6–8]^ Although RA and secondary ITP are common clinical diseases, we found that effective treatment of ITP is rarely reported in patients with RA in China. Therefore, we reviewed the changes of drug therapy and laboratory tests in this patient, providing an important reference for the treatment of ITP with RA.

The pathogenesis of ITP is related to immune intolerance to autoantigen. Platelet destruction mediated by autoantibodies or abnormal activation of cellular immunity are the main pathogeneses of ITP. Studies have shown that about 50% to 60% patients of ITP have anti-IgG autoantibodies on the surface of platelets, which can recognize a variety of glycoproteins, such as GPIIb/IIIa and GPIb/IX.^[9,10]^ These anti-platelet antibodies are recognized by macrophages. FcyIIA on macrophages can bind to anti- glycoproteins on platelets, which can activate spleen tyrosine kinase (Syk) and further lead to phagocytic destruction of platelets. Animal experiments suggest that high dose of IVIG is effective against thrombocytopenia induced by anti-GPIIb/IIIa, but has no obvious effect against thrombocytopenia induced by anti-GPIb/IX.^[11]^

Our patient was an elderly woman with a 5-year history of RA, and the DAS-28 indicated moderate disease activity, which suggested a possible abnormal activation of the immune system. On admissions, she did not respond to different first-line therapies including glucocorticoids and IVIG. She also did not respond to a low dose of EPAG combined with first-line therapy. EPAG was an oral thrombopoietin receptor agonist approved for the treatment of thrombocytopenia in adults with chronic immune thrombocytopenia.^[12]^ Rituximab, an anti-CD20-directed cytosolic monoclonal antibody, inhibited B cell autoantibody production. It could reverse T cell abnormalities in patients who have responded to therapy.^[13]^ Previous studies showed that clinical symptoms in patients with chronic immune thrombocytopenia might not relieve by using EPAG alone.^[14]^ Rituximab was used in a number of clinical treatments, including hematological malignancies, autoimmune diseases, and organ transplantation. A prospective cohort study of children with severe chronic ITP showed a median response time of 1 week and duration of at least 4 weeks to rituximab treatment in patients whose initial platelet count was less than 30 × 10^9^/L.^[15]^ Unlike previous cases, we explained the costs and side effects of second-line treatment to the patient, then she was first given a small dose (25 mg) of oral EPAG for 3 days, followed by rituximab 100 mg. 3 days after the first cycle (rituximab 100 mg weekly combined with EPAG 25 mg, ciclosporin 150 mg and prednisone 40 mg daily), platelet counts and petechiae had significantly improved. 3 days after the second cycle, platelet counts and petechiae were all resolved. The above treatment suggested that for patients with RA complicated with refractory ITP, the use of a smaller dose of EPAG alone might not improve symptoms, but early treatment combined with rituximab could improve platelet counts and clinical symptoms.^[16]^ Meanwhile, the response time of platelet counts was shorter than hematologic patients. It is suggested that for the treatment of RA with refractory ITP, early combination of second-line drugs should be used, and the dosage of EPAG should be appropriately reduced.

We are actively treating the patient's disease while also looking for laboratory indicators that are clinically applicable, inexpensive, and highly sensitive. Reticular platelets are newly released from bone marrow and are young platelets rich in RNA. The proportion and number of reticulated platelets in blood can reflect the platelet formation ability in bone marrow. In this study, we used XE-pro IPF Master software of Sysmex XE-2100 hematology analyzer to count platelets and IPF at the same time. This study showed that when the patient did not respond to first-line therapy, persistently low platelet counts and high IPF suggested increased destruction of mature platelets and accelerated PLT generation of megakaryocytes in bone marrow. After treatment with rituximab combined with EPAG, destruction of mature platelets was reduced, and a part of reticular PLT with myelodysplasia had become mature PLT. Laboratory examination showed that IPF decreased and platelet counts increased. It has been shown that changes of IPF increased before platelet count in patients with non-hematologic tumors after chemotherapy.^[17]^ It was suggested that IPF provided a possibility for dynamic monitoring in clinic, and had a certain value for guiding clinical medication in tumor treatment. IPF in children with ITP was significantly higher than that in patients with aplastic anemia and normal controls, suggesting that IPF could effectively reflect the hyperplasia of megakaryocytes in bone marrow and reduce the pain caused by bone marrow puncture.^[18]^ In this study, the proportion of CD41, CD61, and CD42b in platelet did not change significantly during the whole treatment cycle, while IPF and platelet counts changed significantly, suggesting that changes in IPF and platelet counts were superior to flow cytometry, which may be a new indicator to help predict the effectiveness of drug therapy for secondary ITP in RA.

In conclusion, for patients with refractory ITP in RA, early treatment of rituximab combined with EPAG is beneficial to the remission of patients’ disease. In terms of disease dynamic monitoring, IPF may be an auxiliary indicator for predicting efficacy, but its significance needs further study.

## Author contributions

**Conceptualization:** Chengliang Yuan.

**Data curation:** Naidan Zhang.

**Formal analysis:** Naidan Zhang.

**Funding acquisition:** Naidan Zhang, Chengliang Yuan.

**Investigation:** Naidan Zhang, Xiao Bao.

**Methodology:** Chaixia Ji, Chengliang Yuan.

**Project administration:** Naidan Zhang, Chengliang Yuan.

**Resources:** Xiao Bao.

**Software:** Naidan Zhang.

**Supervision:** Xiao Bao.

**Writing – original draft:** Naidan Zhang.

**Writing – review & editing:** Chengliang Yuan.
